# Physical Signs in the Early Stages of Pregnancy

**Published:** 1898-02

**Authors:** A. R. Martin


### PHYSICAL SIGNS IN THE EARLY STAGES OF PREGNANCY.

Every practicing physician is called upon, sooner or later, to express an opinion in the earliest stages of a suspected pregnancy. All authors agree that a positive diagnosis can not be made until fœtal heart sounds, fœtal movements, or a Braxton-Hicks sign can be elicited. The signs the writer is about to enumerate can be felt only by skillful manipulation, patience and perseverance. Taken then in connection with the general probable signs and symptoms, a fairly safe diagnosis may be made; infallibility, however, is not maintained.

For general consideration, we will divide them under the head of size, shape and consistency:

I. *Size.*—The pregnant uterus increases in size according to the growth of the ovum. This increase is uniform until the third month, after which the size of the fundus is a fairly safe index to the probable stage of pregnancy. During the first eight weeks, the increase in size is very small. Pathological as well as physiological conditions may cause this increase and variation in size. We may enumerate such as submucous myomata; the soft, deep-seated myoma; chronic metro-endometritis, especially the hyperplastic glandular variety; sarcoma; congestion of the organ due to displacement, in the early stages, besides the changes in the organ upon palpation. Then it contracts almost immediately. This latter condition is quickest during menses, slowest when impregnated, under repeated successive examinations. This contraction is succeeded by turgescence, hardening and increase in size. This is so marked during examinations at clinics, that the organ may attain to double the size found during the first examination. Besides all this, we have to overcome subjective difficulties, inasmuch as a hard body seems larger and a soft body smaller to our sense of touch. It is obvious, then that very little can be learned from the size of the organ.

II. *Shape.*—The unimpregnated uterus is pear-shaped, flattened antero-posteriorly, transverse diameter slightly larger than antero-posterior. In the pregnant uterus, the transverse diameter increases more markedly than the antero-posterior. This is contrary to the rule in metritis and intra-uterine tumors. Anterior rotation may become so marked that the fundus partially enters the inferior conjugate diameter. This rotation draws up the anterio-vaginal wall and makes it more tense, and the cervix becomes shorter.

III. *Consistency.*—This is our chief diagnostic point, since it gives us the most important signs. The peculiar consistency or bogginess (if you please) must be felt to be appreciated. Of pathological conditions it resembles most the hyperplastic glandular endometritis or the old fungous endometritis of

Olshauzen, soft myomata, a fairly well-developed sarcoma and the congestion found in the early stage of an extremely retroverted uterus. The sensation is as if the finger would easily penetrate the organ, still there is no pitting or depression left. The sensation is more real if the patient is examined in the standing position. The greatest difference is found at the sites of cellular tissue, supra-vaginal or at site of sacro-uterine ligaments and at junction of body and tubes, Hegar's sign, which the writer wishes to call especial attention to, since it is mistaken by some authors. This is a soft, parchment-like sensation imparted to the touch, and should be made with the thumb in the vagina and finger in the rectum, with downward pressure externally, and is found at junction of supra-vaginal portion of cervix with fundus, and decreases downward toward end of cervix. The same soft, parchment-like condition is found at the junction of tubes with the fundus. This peculiar softening and thinness, at the sites mentioned, gives the examining finger the impression as if the organ had lost all its former connections in the pelvis, and when found is pathognomonic of pregnancy.

Then, finally, we find a difference in the walls of the pregnant organ. Most often the anterior wall is more boggy than the posterior, probably due to site of placental implantation. It is almost impossible to demonstrate the conditions enumerated to a class of students because of the contractions of the organ almost immediately it is secured between the fingers. The writer has found it necessary to allow a lapse of about fifteen minutes to supervene, until the organ relaxes, and the student can find the condition demonstrated.—DR. A. R. MARTIN, in *Denver Med. Times.—Atlantic Med. Weekly.*

